# Personalised Normative Feedback for Preventing Alcohol Misuse in University Students: Solomon Three-Group Randomised Controlled Trial

**DOI:** 10.1371/journal.pone.0044120

**Published:** 2012-09-12

**Authors:** Maria T. Moreira, Reza Oskrochi, David R. Foxcroft

**Affiliations:** 1 Instituto Superior de Saúde do Alto Ave, Póvoa de Lanhoso, Braga, Portugal; 2 Medical Statistics Programme, Oxford Brookes University, Oxford, Oxfordshire, United Kingdom; 3 Department of Psychology, Social Work and Public Health, Oxford Brookes University, Oxford, Oxfordshire, United Kingdom; University of Pennsylvania, United States of America

## Abstract

**Background:**

Young people tend to over-estimate peer group drinking levels. Personalised normative feedback (PNF) aims to correct this misperception by providing information about personal drinking levels and patterns compared with norms in similar aged peer groups. PNF is intended to raise motivation for behaviour change and has been highlighted for alcohol misuse prevention by the British Government Behavioural Insight Team. The objective of the trial was to assess the effectiveness of PNF with college students for the prevention of alcohol misuse.

**Methodology:**

Solomon three-group randomised controlled trial. 1751 students, from 22 British Universities, allocated to a PNF group, a normal control group, or a delayed measurement control group to allow assessment of any measurement effects. PNF was provided by email. Participants completed online questionnaires at baseline, 6- and 12-months (only 12-months for the delayed measurement controls). Drinking behaviour measures were (i) alcohol disorders; (ii) frequency; (iii) typical quantity, (iv) weekly consumption; (v) alcohol-related problems; (vi) perceived drinking norms; and (vii) positive alcohol expectancies. Analyses focused on high-risk drinkers, as well as all students, because of research evidence for the prevention paradox in student drinkers.

**Principal Findings:**

Follow-up rates were low, with only 50% and 40% responding at 6- and 12-months, respectively, though comparable to similar European studies. We found no evidence for any systematic attrition bias. Overall, statistical analyses with the high risk sub-sample, and for all students, showed no significant effects of the intervention, at either time-point, in a completed case analysis and a multiple imputation analysis.

**Conclusions:**

We found no evidence for the effectiveness of PNF for the prevention of alcohol misuse and alcohol-related problems in a UK student population.

**Registration:**

Controlled-Trials.com ISRCTN30784467

## Introduction

During university or college years, students can escalate their alcohol use to dangerous levels [1], and student alcohol consumption levels are typically higher than their non-university peers [2]–[6]. Heavy alcohol consumption amongst students impacts on individuals, educational institutions, and society [7], [8]: excessive drinking behaviour amongst students is linked with a range of alcohol related problems, including injuries, unprotected sex, violence, car accidents, and health problems [9], resulting in a significant economic burden for health systems [10].

Social normative feedback is a prevention intervention based on the fact that young people tend to overestimate alcohol consumption amongst their peer group resulting in motivation, or “peer pressure” to drink more to catch up with their peers and be normal. Social norms theory [11] suggests that correcting this misperception will lead young people to attenuate their drinking behaviour. Brief personalised normative feedback (PNF) interventions focus on an individual's own drinking behaviour, providing factual details about personal drinking levels and patterns in comparison with norms for drinking in similar aged peer groups, alongside more general information about alcohol risk and harms [12]–[15]. PNF is intended to raise motivation for behaviour change [16], [17] and has been highlighted as a promising intervention by the British Government Behavioural Insight Team [18], [19]. A recent Cochrane review [20] found some limited evidence for the effectiveness of computer-based PNF in University students though questions remain about the generalizability of these findings to other countries, including the U.K. [21], [22]. There is also a question about whether PNF is effective over and above the simple alcohol screening/assessment test that itself raises awareness about alcohol consumption [23].

The main objective of this study was to examine the effectiveness of computer-based PNF, compared with (i) screening/assessment only, for reducing alcohol-related problems in first and second year UK university undergraduate students. A further consideration is whether PNF would be more effective when used with all students, rather than just those identified as being at higher risk, because of the prevention paradox [24]. The Harvard College Alcohol study of more than 49,000 U.S. students [25] found that most alcohol-related harms arise from those who are not higher risk drinkers, and this finding is supported by evidence from other countries [26]. Therefore, a second objective was to assess the effectiveness of whole population PNF versus PNF for high-risk drinkers only.

## Methods

The protocol for this trial and supporting CONSORT checklist are available as supporting information; see Checklist S1 and Protocol S1.

### Design

The study utilised a Solomon three group randomised controlled trial design [27], with one intervention and two control groups to control separately for intervention and for measurement effects. A more conventional Solomon four group design was not possible given that the intervention required baseline information in order to personalise feedback to each participant. The International Standard Randomised Controlled Trial Number for this study is: ISRCTN30784467. The intervention group received brief personalised normative feedback (PNF) within a few weeks of completing the baseline assessment, which comprised an online questionnaire with demographic questions and an assessment of drinking behaviour. The main control group undertook a baseline alcohol assessment, and the delayed control group only provided demographic details at baseline. The intervention and main control group were followed up at 6- and 12-months, and the delayed control group at 12-months only.

### Participants

Eligible participants were undergraduate university students (first and second year students; academic year 2008/9) from U.K. universities, recruited through university information systems (posters, email messages, bulletin boards) and through online social networking sites. Participants were incentivised to respond through entry into a prize draw, with prizes including games consoles, mp3 players and store vouchers.

### Ethics

Ethics approval for this study was provided by Oxford Brookes University Research Ethics Committee (UREC No. 2006/28). Participants gave their informed consent to participate by completing the web-based questionnaire.

### Intervention

The brief personalised normative feedback (PNF) emailed to each participant in the intervention group one to two weeks post-baseline comprised the results of their drinking behaviour assessment compared with, in an easy to read graphical format, average levels of drinking amongst their student peer group. The feedback also provided general information about alcohol and how it might affect them at their current drinking levels, including how long it could take to return to a zero blood alcohol level after a typical drinking occasion. Information was also provided on the money that they might be spending annually on alcohol and also the calories they might be consuming at their current drinking levels. Details of recommended sensible drinking levels were also provided. The composition of the PNF was informed by online normative feedback provided by the Centre for Addiction and Mental Health (CAMH), Toronto [28], and the Brief Alcohol Screening and Intervention for College Students (BASICS) [29] programme. A specimen feedback is available to download [30].

### Objectives

The main objective of the trial was to assess the effectiveness of PNF with risky drinking college students for the prevention of alcohol misuse. A second objective was to assess the relative effectiveness of whole population (universal) versus screening and brief intervention (SBI; targeted) normative feedback in reducing alcohol related problems.

### Outcomes

A copy of the online questionnaire is available for download [30]. Baseline demographic questions asked about university year, age, gender, living arrangements and weight.

Respondents completed the Alcohol Use Disorders Identification Test (AUDIT) which is a 10-item scale with good validity that is designed to assess hazardous and harmful drinking [31]. The AUDIT score was specified as the primary outcome variable. In another study we have shown that a small change in AUDIT score may have an important impact on population levels of alcohol disorders [32]. The AUDIT scale is scored between 0 and 40, with a higher score indicating higher levels of drinking.

Frequency of alcohol consumption was assessed with one question asking how often the respondent drank, with responses ‘Never’, ‘1–2 per year’, ‘Once a Month’, ‘Twice a Month’, ‘Once a Week’, ‘Twice a Week’, or ‘Daily’. Responses were calculated as number of drinking days per month; for example ‘Never’ scored 0, ‘Once a Week’ scored 4.5, and ‘Daily’ scored 30.

Usual quantity of alcohol consumption was assessed with one question asking how many drinks/units a respondent usually consumed on a drinking occasion, with responses 0, 1, 2, 3, 4, 5, 6, or 7 or more.

Respondents also completed a drinking diary about a “typical week”, where they indicated the number of drinks/units they usually drink on each day of the week, and this was used to calculate the number of units consumed each week. Respondents were then categorised according to whether they were drinking more than 14 units a week for women and more than 21 units a week for men [33]. In the analysis, respondents were scored 1 if they were over these levels, otherwise 0.

Young people who engage in one problem behaviour (e.g. alcohol misuse) are also more likely to engage in other problem behaviours [34]. Therefore, other problems were measured in a newly-developed self-reported scale with nine possible problems, listed on a yes/no scale: 1. Blackout or memory lapse; 2. Been embarrassed by your actions; 3. Been in a fight; 4. Engaged in unprotected sex; 5. Missed a lecture/class; 6. Required emergency medical treatment; 7. Sustained an injury; 8. Trouble with local or campus authorities; 9. Received unwanted sexual advances. Responses were summed to provide an alcohol-related problems score (range 10 to 20 with a higher score indicating more problems). The internal consistency for this scale was alpha = 0.71.

Perceived norms were measured using an adaptation of the two versions of the Drinking Norms Rating Form [35]. We calculated normative misperception from two variables: the number of alcoholic drinks (“yourself” and “students in your year”) respondents felt were on average consumed at parties or social events. The difference between “yourself” and “students in your year” ranged between −7 and +5, with a negative score indicating that respondents thought that other students were drinking more.

Positive alcohol expectancies were measured using an abbreviated form of the Alcohol Expectancies Questionnaire for Adolescents (AEQ-A) [36]. This self-report questionnaire assesses positive expectancies related to alcohol consumption (social changes, cognitive improvement, sexual enhancement and relaxation) with 21 statements and a true/false answer format. Scores ranged between 0 and 21, with a higher score indicating more positive expectancies.

Several measures were included in the questionnaire to be used as covariates in analyses, including demographic factors (age, gender) as well as baseline consumption. Social desirability responsiveness was also assessed using the short form of the Marlowe Crown scale 2 [37].

### Sample Size

We needed an achieved sample size of 900 hazardous drinkers (150 per gender per group), based on effect size estimates of a mean difference in AUDIT score of 1.9 (s.d. = 6) for females [38], and power = .9 and α = .05 (2-tailed tests). Taking account of known prevalence rates for hazardous drinkers in this population, and with cautious estimates for participation and attrition rates, we aimed to recruit 4000 students.

### Randomization – sequence generation

Participants who provided informed consent were individually randomised to intervention or control groups via concealed centrally-allocated computer generated random numbers, with a 1∶1∶1 allocation ratio, without stratification or blocking on any variables.

### Randomization – allocation concealment

The computer-based randomisation ensured that researchers and participants were not aware of allocated group.

### Randomization – implementation

Participants were recruited via a web site that provided trial information and enabled informed consent. Once consent was given, participants were randomized by computer. There was no direct human involvement in the randomization process.

### Blinding

At randomisation participants were not aware of the nature of the intervention, but full participant blinding was not possible. Similarly, the postgraduate researcher who emailed the standardised PNF to the intervention group participants was not blind to group allocation. All questionnaires were completed remotely via web-based forms, and so outcome assessment was blinded.

### Statistical Methods

Data from the remotely completed online questionnaires were automatically entered and stored in a web based server. At the end of the trial a computer science specialist de-encrypted the data and all personal identifying details were removed from the dataset.

All analyses were intention-to-treat (ITT) and all participants were followed up regardless of their compliance with the intervention. Analyses were performed on the full sample (all students) and also with a higher risk sub-sample to test the prevention paradox prediction. Higher risk drinkers were those who, at baseline, scored 8 or more on the AUDIT scale (the cut-off for hazardous drinking) and also drank more than the recommended weekly consumption limits.

The collected scores about the “Frequency of drinking” and the “Quantity of drinking” were analysed with a mixing distribution of the Poisson regression with a Gamma mixture for panel data using the Stata 10.1 (StataCorp, College Station, Texas). For the proportions of students exceeding 21 units a week (men) or 14 units a week (women), we used generalized linear mixed models with the xtlogit procedure. All other scores (Audit, Alcohol-related Problems, Drinking Norms and Positive Expectancies) were analyzed with linear/log-linear regression using xtmixed (StataCorp). All models included a random intercept to account for clustering within participant (same university) as well as fixed effects for group, follow-up assessment and their interaction. The interactions were removed from the analysis due to insignificant effects. The results are presented as relative risks, odds ratios and difference in regression coefficients, respectively.

We fitted two different random effect models to assess an intention-to-treat hypothesis. In the first random effects model we treated all missing values as missing at random (MAR). Random effects models [29], [30] allow the incorporation of all participants with at least 1 follow-up observation. This model will yield unbiased estimates of the treatment effect under the assumption that values are missing at random. In the second random effects model we carried out a sensitivity analysis using multiple imputation. A chained equation imputation model was fittedsimultaneously for all outcomes as well as baseline AUDIT score, age, and sex, to create imputed complete data sets. Values were imputed for all participants, even for those with no post-baseline data. The following assumptions [39] guided the multiple imputation: 1. all randomised participants were followed-up even if they did not participate in the intervention (i.e. they withdrew from the allocated treatment); 2. We performed a main analysis of all observed data that are valid under a plausible assumption about the missing data; and 3. We performed sensitivity analyses to explore the effect of departures from the assumption made in the main analysis.

## Results

### Participant flow

**Figure 1:**
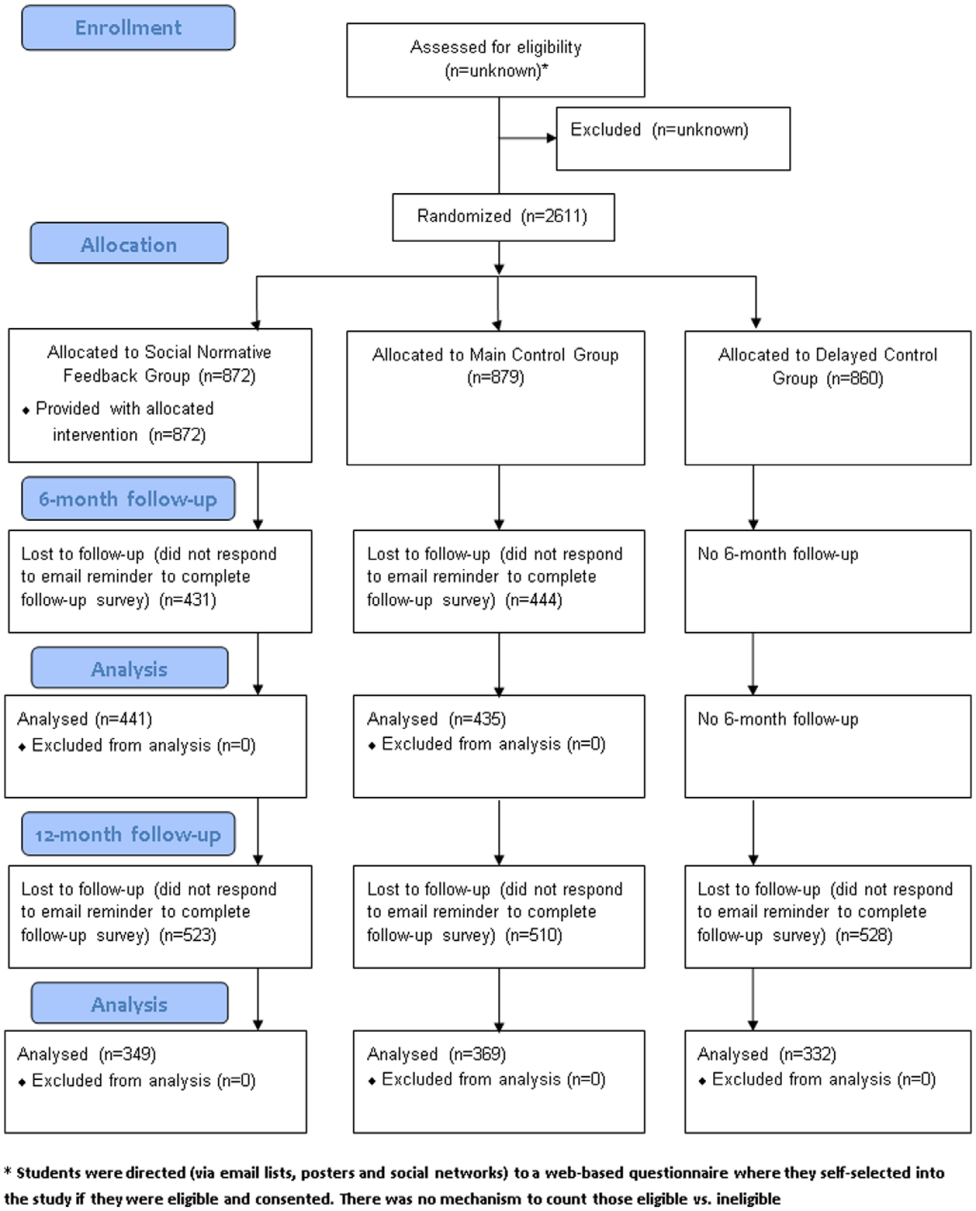
CONSORT Participant Flow Diagram.

The sample at baseline (N = 2611) consisted of first and second year students enrolled in UK universities. Recruitment, follow-up and attrition are described in Figure 1. Follow-up rates were low, with only 50% responding at 6-months and only 40% responding at 12-months. We were not able to collect information about reasons for non-completion of follow-up questionnaires.

### Recruitment

Students were recruited into the study at the beginning of the academic year 2007/8, and followed up for 6- and 12-months.

### Baseline data

Sample characteristics are described in Table 1. Table 2 describes participants lost to follow-up, with very similar rates in both intervention and the main control groups, suggesting that there was no differential or systematic bias in attrition.

**Table 1:** Baseline Demographic Characteristics and Alcohol Use by Group.

High Risk sub-sample	Intervention (N = 591)	Main Control (N = 596)	Delayed Control
Female Sex N (%)	335 (56.7)	351 (58.9)	*a*
Age, N (%)			
17–19	365 (61.8)	384 (64.4)	*a*
20–24	199 (33.7)	190 (31.9)	*a*
>25	27 (4.6)	22 (3.7)	*a*
Living arrangement %			
Student hall of residence	291(49.2)	309 (51.8)	*a*
Rented accommodation	244 (41.3)	237 (39.8)	*a*
Home with parents	38 (6.4)	33 (5.5)	*a*
Other	18 (3.0)	17 (2.9)	*a*
AUDIT Score, mean (SD)	14.77 (5.87)	14.41 (5.56)	*a*
No. of standard drinks per typical drinking occasion, mean (SD)	6.97 (1.29)	6.84 (1.30)	*a*
Alcohol-related problems, mean (SD)	16.43 (1.93)	16.50 (1.97)	*a*

Full Sample (all students)	Intervention (N = 872)	Main Control (N = 879)	Delayed Control (N = 860)
Female Sex, N (%)	541 (62.0)	536 (61.0)	515 (60.0)
Age, N (%)			
17–19	497 (57.0)	547 (62.2)	513 (59.7)
20–24	320 (36.7)	281 (32.0)	300 (34.9)
>25	55 (6.3)	51 (5.8)	47 (5.5)
Living arrangement, N (%)			
Student hall of residence	405 (46.4)	439 (49.9)	441 (51.3)
Rented accommodation	366 (42.0)	342 (38.9)	331 (38.5)
Home with parents	65 (7.5)	69 (7.8)	66 (7.7)
Other	36 (4.1)	29 (3.3)	22 (2.6)
AUDIT Score, mean (SD)	11.25 (7.15)	11.00 (6.86)	*a*
No. of standard drinks per typical drinking occasion, mean (SD)	5.97 (2.1)	5.90 (2.1)	*a*
Alcohol-related problems, mean (SD)	17.32 (2.14)	17.37 (2.16)	*a*

*a: Figures not available as no alcohol data was collected for the Delayed Control group at baseline; only demographic information was collected.*

**Table 2:** Unavailable for follow-up analysis.

	Missing at 6 months		Missing at 12 months		Missing at both time points	
High Risk sub-sample	Intervention N = 317	Control N = 320	Intervention N = 388	Control N = 355	Intervention N = 282	Control N = 268
Female, N (%)	166 (52.4)	187 (58.4)	207 (53.4)	198 (55.8)	147 (52.1)	152 (56.7)
Age, N (%)						
17–19	194 (61.2)	207 (64.7)	239 (61.6)	228 (64.2)	171 (60.6)	173 (64.6)
20–24	109 (34.4)	106 (33.1)	136 (35.1)	118 (33.2)	100 (35.5)	90 (33.6)
>25	14 (4.4)	7 (2.2)	13 (3.4)	9 (2.5)	11 (3.9)	5 (1.9)
AUDIT score at baseline, Mean (SD)	15.14 (5.93)	15.11 (5.80)	15.17 (5.97)	15.01 (5.72)	15.32 (6.10)	15.26 (5.77)
**Full Sample (all students)**	N = 431	N = 444	N = 523	N = 510	N = 379	N = 382
Female, N (%)	247 (57.3)	261 (58.8)	298 (57.0)	290 (56.9)	213 (56.2)	220 (57.6)
Age, N (%)						
17–19	246 (57.1)	282 (63.5)	301 (57.6)	312 (61.2)	215 (56.7)	241 (63.1)
20–24	163 (37.8)	145 (32.7)	197 (37.7)	175 (34.3)	145 (38.3)	126 (33.0)
>25	22 (5.1)	17 (3.8)	25 (4.8)	23 (4.5)	19 (5.0)	15 (3.9)
AUDIT score at baseline, Mean (SD)	12.16 (7.22)	12.04 (7.08)	12.30 (7.18)	11.71 (7.02)	12.40 (7.32)	11.95 (7.13)

### Numbers analysed

Intention to treat was applied in all analyses. In the completed case analyses (i.e. follow-up respondents only) for high risk drinkers 104/116 universities and 550/1187 individuals were followed up and analysed at 6-months, and 97/116 universities and 444/1187 individuals at 12-months. In the full population analyses 111/122 universities and 876/1751 individuals were followed up and analysed at 6-months, and 107/122 universities and 718/1751 individuals at 12-months.

### Outcomes and estimation

We have not included the delayed control group in attrition assessment or statistical analysis of effects because there were no statistical differences between the main control and the delayed control groups at 12-month follow-up for any drinking behaviour measures (frequency of drinking, χ^2^ = 6.29, df = 6, p = 0.39; usual quantity of alcohol, t = 0.075, df = 699, p = 0.94; AUDIT, t = 0.63, df = 699, p = 0.53; alcohol-related problems, t = −0.181, df = 699, p = 0.86; perceived drinking norms, t = −0.609, df = 699, p = 0.54). This analysis indicates that there was no effect on drinking behaviour of the baseline measures and questions about alcohol use and problems.

Six- and twelve-month follow-up analysis in the high risk sub-sample showed no effects of the intervention, at either time-point (Tables 3 and 4) and in both the completed case analysis and the multiple imputation analysis, for most outcomes. Only one outcome measure, weekly drinking, showed a significant effect at 6-months with an odds ratio (OR) of 0.417 (95% CI 0.223, 0.781), but this had disappeared by 12-months (OR = 0.710, 95% CI 0.435, 1.160). The 12-month significant effect remained in the multiple imputation analysis, but this should be regarded with caution given the very high attrition rates. Moreover, this one effect should not be over-interpreted given the consistent pattern of no effect across most outcome measures and time points.

**Table 3:** Summary statistics for high risk sub-sample.

	Intervention			Control		
	Mean	SD	N	Mean	SD	N
**Frequency of Drinking per month**						
6 months	8.90	7.41	274	9.43	7.83	276
12 months	8.06	6.86	203	8.70	7.00	241
**Quantity of drinking per Occasion**						
6 months	5.44	1.42	274	5.48	1.56	276
12 months	5.31	1.52	203	5.43	1.50	241
**Weekly drinking (proportion)**						
6 months	.61	.49	274	.66	.47	276
12 months	.42	.49	184	.49	.50	205
**AUDIT score**						
6 months	12.05	5.64	274	11.81	5.58	276
12 months	11.37	5.55	203	11.32	5.55	241
**Alcohol-related Problems**						
6 months	16.83	1.97	274	16.90	2.04	276
12 months	17.15	2.04	203	17.31	2.05	241
**Drinking Norms**						
6 months	−.376	1.31	274	−.464	1.33	276
12 months	−.547	1.28	203	−.610	1.31	241
**Positive Expectancies**						
6 months	11.88	2.80	274	12.15	2.78	276
12 months	11.79	2.61	203	12.07	2.86	241

**Table 4:** High Risk sub-sample analysis results.

	Without Imputation		With multiple imputation	
N	Universities	Observation	Universities	Observation
baseline	116	1187	116	1187
After 6 month	104	550	116	1187
After 12 months	97	444	116	1187

Frequency of Drinking^1^	RR	95% CI	P-value	RR	95% CI	P-value
baseline	0.990	(0.945, 1.038)	0.682	0.990	(0.945, 1.038)	0.682
After 6 month	0.990	(0.922, 1.063)	0.793	0.998	(0.950, 1.049)	0.945
After 12 months	0.975	(0.900, 1.057)	0.547	1.007	(0.973, 1.059)	0.780
**Quantity of Drinking** 2						
baseline	1.049	(0.919, 1.199)	0.477	1.049	(0.919, 1.199)	0.477
After 6 month	0.980	(0.819, 1.174)	0.828	0.981	(0.868, 1.109)	0.764
After 12 months	0.968	(0.805, 1.165)	0.735	0.961	(0.847, 1.089)	0.531
**Weekly Drinking** 3	**OR**			**OR**		
baseline	1.250	(0.912, 1.714)	0.164	1.250	(0.912, 1.714)	0.164
After 6 month	0.417	(0.223, 0.781)	0.006	0.380	(0.251, 0.574)	0.000
After 12 months	0.710	(0.435, 1.160)	0.172	0.619	(0.475, 0.808)	0.000
**AUDIT Score** 4	**Beta**			**Beta**		
baseline	0.718	(0.009, 1.428)	0.047	0.718	(0.009, 1.428)	0.047
After 6 month	−0.292	(−0.929, 0.345)	0.369	−0.067	(−0.511, 0.377)	0.766
After 12 months	−0.363	(−1.157, 0.430)	0.369	−0.228	(−0.749, 0.293)	0.392
**Alcohol-related Problems** 4	**Beta**			**Beta**		
baseline	−0.132	(−0.374, 0.109)	0.282	−0.132	(−0.374, 0.109)	0.282
After 6 month	0.045	(−0.202, 0.292)	0.723	0.120	(−0.065, 0.306)	0.204
After 12 months	−0.021	(−0.331, 0.289)	0.895	−0.038	(−0.225, 0.149)	0.690
**Drinking Norms** 4	**Beta**			**Beta**		
baseline	0.159	(0.003, 0.314)	0.045	0.159	(0.003, 0.314)	0.045
After 6 month	0.022	(−0.164, 0.209)	0.813	−0.044	(−0.171, 0.084)	0.503
After 12 months	0.024	(−0.195, 0.242)	0.832	0.029	(−0.105, 0.162)	0.673
**Positive Expectancies** 4	**Beta**			**Beta**		
baseline	0.004	(−0.309, 0.318)	0.979	0.004	(−0.309, 0.318)	0.979
After 6 month	−0.136	(−0.495, 0.222)	0.457	0.006	(−0.238, 0.250)	0.962
After 12 months	−0.210	(−0.681, 0.260)	0.381	−0.139	(−0.458, 0.179)	0.392

1 Random effect Poisson (negative binomial) model;

2 Poisson population average model (Generalised linear mixed model);

3 Random Effect Logistic model (Generalised linear mixed model);

4 Random effect linear model (linear mixed model).

In the full sample analysis there was a similar pattern of no effect of the intervention (Tables 5 and 6) at either time-point and in both the completed case analysis and the multiple imputation analysis. Again, only weekly drinking had a significant effect at six-months (OR = 0.440, 95% CI 0.245, 0.788), but not at 12-months (OR = 0.770, 95% CI 0.495, 1.197). Overall, there is a consistent pattern of no effect across most outcome measures and time points.

**Table 5:** Summary statistics for full sample (all participants).

	Intervention			Control		
	Mean	SD	N	Mean	SD	N
**Frequency of Drinking per month**						
6 months	6.90	6.83	441	7.25	7.28	435
12 months	6.04	6.10	349	6.73	6.66	369
**Quantity of drinking per Occasion**						
6 months	4.44	2.05	441	4.48	2.15	435
12 months	4.32	2.09	369	4.46	2.09	369
**Weekly drinking (proportion)**						
6 months	0.381	0.49	441	0.418	0.49	435
12 months	0.267	0.44	315	0.318	0.47	327
**AUDIT score**						
6 months	8.90	6.25	441	8.83	6.18	435
12 months	8.13	5.97	349	8.59	6.08	369
**Alcohol-related Problems**						
6 months	17.77	2.08	441	17.77	2.08	435
12 months	18.05	2.01	349	18.02	2.01	369
**Drinking Norms**						
6 months	−1.240	1.88	441	−1.317	1.92	435
12 months	−1.352	1.80	349	−1.377	1.84	369
**Positive Expectancies**						
6 months	11.16	3.12	441	11.54	2.95	435
12 months	11.14	2.94	349	11.61	3.03	369

**Table 6:** Full sample (all participants) analysis results.

	Without Imputation		With multiple imputation	
N	Universities	Observation	Universities	Observation
baseline	122	1751	122	1751
After 6 month	111	876	122	1751
After 12 months	107	718	122	1751

Frequency of Drinking^1^	RR	95% CI	P-value	RR	95% CI	P-value
After 6 month	0.992	(0.935, 1.054)	0.805	0.987	(0.945, 1.031)	0.568
After 12 months	0.982	(0.918, 1.050)	0.594	0.980	(0.936, 1.026)	0.392
**Quantity of Drinking** 2						
After 6 month	0.977	(0.923, 1.034)	0.421	0.997	(0.957, 1.039)	0.896
After 12 months	0.994	(0.933, 1.059)	0.856	0.986	(0.947, 1.026)	0.480
**Weekly Drinking** 3	**OR**			**OR**		
After 6 month	0.440	(0.245, 0.788)	0.006	0.410	(0.276, 0.610)	0.000
After 12 months	0.770	(0.495, 1.197)	0.246	0.820	(0.637, 1.055)	0.123
**AUDIT Score** 4	**Beta**			**Beta**		
After 6 month	−0.244	(−0.676, 0.187)	0.267	−0.075	(−0.384, 0.233)	0.633
After 12 months	−0.209	(−0.740, 0.321)	0.439	−0.090	(−0.445, 0.264)	0.618
**Alcohol-related Problems** 4	**Beta**			**Beta**		
After 6 month	0.082	(−0.107, 0.271)	0.396	0.119	(−0.008, 0.246)	0.066
After 12 months	0.020	(−0.191, 0.231)	0.851	0.104	(−0.031, 0.239)	0.132
**Drinking Norms** 4	**Beta**			**Beta**		
After 6 month	−0.022	(−.187, 0.144)	0.797	−0.041	(−0.075, 0.158)	0.489
After 12 months	0.055	(−0.167, 0.277)	0.628	0.097	(−0.044, 0.238)	0.179
**Positive Expectancies** 4	**Beta**			**Beta**		
After 6 month	−0.112	(−0.410, 0.185)	0.458	−0.029	(−0.256, 0.198)	0.800
After 12 months	−0.197	(−0.562, 0.168)	0.290	−0.158	(−0.390, 0.074)	0.182

1 Random effect Poisson (negative binomial) model;

2 Poisson population average model (Generalised linear mixed model);

3 Random Effect Logistic model (Generalised linear mixed model);

4 Random effect linear model (linear mixed model).

### Ancillary analyses

No ancillary analyses were undertaken.

### Adverse events

No adverse events were reported.

## Discussion

We have found that a UK web-based personalised normative feedback (PNF) intervention did not motivate students to reduce their alcohol intake: students who received the brief personalised PNF intervention did not have lower AUDIT scores, drink less alcohol or have fewer alcohol related problems than control group students at 6- or 12-month follow-ups. Our results fail to replicate the findings of the New Zealand, Australian and U.S. trials of brief, web-based, social normative feedback [40]–[42]. But our results are generally in agreement with other work from the UK and Sweden, where randomised trials aimed at assessing the effectiveness of an electronic web-based personalised feedback intervention have found little or no effect for most outcome measures [43]–[46].

### Interpretation

The innovative design and sampling aspects of this study: the Solomon three-group design to test for measurement effects, and the whole sample and higher risk sub-sample analyses to test the prevention paradox prediction, were illuminating. We found no evidence that simply asking people about their drinking behaviour, in the absence of any intervention, led to any change in behaviour. Similarly, we found no evidence that a population wide intervention was more effective than a targeted intervention with a higher risk sub-sample of students but, given that we found no effects of the PNF intervention across a range of alcohol outcomes, this is not surprising.

### Generalizability

The reason(s) for the inconsistent findings between our study and some other studies is not clear. One line of thought is that the accumulating evidence on social normative feedback is an example of initially positive research findings ultimately being found to be false [47]. Another possibility is that the UK offers a more challenging context for effectiveness because of local contextual factors that are not present in other countries, for example it may be that the prevalence of excessive drinking behaviours in the UK, and the cultural acceptance of heavier drinking, provides a more hostile environment for success of normative feedback interventions. Alternatively, the intervention we used, based on studies from other countries, may not have been sufficiently developed for the UK population. In this study personalised normative feedback was presented to university students via email with information about the norms for the “average” student in their university, as in previous research [48] where similar proximal referent norms were used. Research has demonstrated that when compared with more distal referents, proximal referents are more effective for preventing student alcohol misuse and related problems [35], [49], [50]. On the other hand, a study in Canada [51] found that the social norm materials that were designed to be the least comparable had the greatest impact, and that Canadian students were more familiar with American drinking norms because of media influence, with television being the most significant medium for the spread of youth culture.

We should also point out that, like many other studies that have reported the effects of brief personalised normative feedback, the intervention contained more than just the normative feedback. We also provided financial feedback, health information and advice on where help can be obtained. The interaction between these different components, and how they are presented to participants, may be important in determining effectiveness. Clues as to the differences in effect across different studies might lie in the differences in intervention content and delivery.

Attrition in this study was lower than in other European research on email- or web-based social normative feedback interventions with university students [44]–[46] though higher than in some Australian or U.S. studies [40], [42]. There is a potential risk to internal validity from the low follow-up rates achieved in our study, although students unavailable for follow-up were similar across groups with regard to sex, age and baseline drinking status, and the multiple imputation sensitivity analysis did not produce any marked or systematic changes in treatment effect sizes or significance. But, due to the low follow-up rates, we cannot absolutely rule out the possibility that differential attrition in relation to unmeasured and uncontrolled confounders could have affected the results.

One possible reason for the low retention rates in our study is that the personalised feedback given in our study was delayed by a few weeks, as we needed to collect and analyse all baseline data to provide a normative comparison dataset for personalised feedback to each participant. It is conceivable that this could have influenced retention rates, but we regard this as unlikely given more immediate feedback was provided in similar studies that had similar or poorer retention rates [44]–[46] and similar findings of limited/no effectiveness.

A more serious issue is in relation to external validity, specifically generalizability [22]. It is hard to see how the sub-set of students that were retained in the study are generally representative of the whole student population, so inferences from sample to the pre-specified study population are problematic. In fact, the low-follow-up rates add to the problem with understanding how generalizable these study results are given the recruitment methods used: only those students who saw the study adverts (via email, student information systems or Facebook) were able to participate and, as the most effective recruitment strategy was Facebook with 78% of all participants, it is unclear how representative these participants are of the general student body. So, one possible explanation for our null results in this study is that we obtained results on a different group of students than other studies that have found significant effects.

On the other hand, this study was a large pragmatic randomised trial with design, sampling, recruitment and follow-up characteristics that are similar to other large European trials [44]–[46]. All these other European trials have had low follow-up rates from those randomised and assessed at baseline, and with similar non-significant effects to our study. An important conclusion to draw out from our study, alongside these other European studies, is that the acceptability and viability of recruiting students into a brief personalised feedback intervention, outside of a university medical centre screening programme [40] or a mandated student alcohol education programme [42], seems to be low.

### Overall evidence

In conclusion, our results show no evidence for the effectiveness of personalised normative feedback for the prevention of alcohol misuse and alcohol-related problems in a UK student population. The applicability of personalised normative feedback to populations and settings that are different from those in the trials whether positive effects have been found is uncertain [22]; moreover an overall conclusion of no effect for this sort of intervention [47] cannot be discounted.

## Supporting Information

Checklist S1 **CONSORT Checklist.**
(DOC)

Protocol S1 **Trial Protocol.**
(PDF)
