# Supplementary material for: Personalised Normative Feedback for Preventing Alcohol Misuse in University Students: Solomon Three-Group Randomised Controlled Trial
Source: PLoS One. 2012 Sep 12;7(9):e44120. doi: 10.1371/journal.pone.0044120 (PMC3440433; doi:10.1371/journal.pone.0044120)
Supplement: Protocol S1 — Trial Protocol. (PDF) [file pone.0044120.s002.pdf]

BMC Public Health

Study protocol

Open Access

The effectiveness of brief personalized normative feedback in reducing alcohol-related problems amongst University students: protocol for a randomized controlled trial

Teresa Moreira and David R Foxcroft*

Address: School of Health and Social Care, Oxford Brookes University, Oxford, OX3 0FL, UK

Email: Teresa Moreira - mtmoreira@brookes.ac.uk; David R Foxcroft* - david.foxcroft@brookes.ac.uk

* Corresponding author

Published: 10 April 2008

BMC Public Health 2008, **8**:113 doi:10.1186/1471-2458-8-113

Received: 13 February 2008
Accepted: 10 April 2008

This article is available from: http://www.biomedcentral.com/1471-2458/8/113

© 2008 Moreira and Foxcroft; licensee BioMed Central Ltd.
This is an Open Access article distributed under the terms of the Creative Commons Attribution License (http://creativecommons.org/licenses/by/2.0), which permits unrestricted use, distribution, and reproduction in any medium, provided the original work is properly cited.

Abstract

Background: Studies have shown that university/college students tend to have an exaggerated view of the quantities of alcohol being consumed by their peers. Making students aware of this misperception may help change behaviour and reduce problem drinking.

Methods/Design: A Solomon Three Group Design will be used. There is one intervention group and two control groups, controlling separately for measurement and for intervention effects. Recruitment, consent, randomisation and data collection are all on-line. The primary outcomes are AUDIT Score, weekly consumption, perceived social norms, and alcohol related problems; secondary outcomes include alcohol expectancies and other health behaviours.

Discussion: This trial will provide information on the effectiveness of an on-line personalized normative feedback intervention for alcohol misuse in university students.

Trial registration: International Standard Randomised Controlled Trial Number: ISRCTN30784467

Background

Alcohol related problems

Around 55,000 young people in Europe died from causes related to alcohol use in 1999 [1,2]. In the UK alcohol-related mortality is increasing compared with many other European countries where rates are declining or unchanged. At the same time, binge drinking rates amongst young people are high in the UK and Holland, and are increasing in the UK where alcohol related violence and crime is a major cause for concern. Therefore an effective prevention programme that has a significant impact on alcohol related problems amongst young people would be very important.

Normative feedback

Normative feedback as an approach to alcohol misuse prevention is based on Social Norming Theory. Initially developed in the United States, this approach relies on changing the attitudes and norms that exist around drinking behaviours, typically on University campuses. The normative feedback approach relies largely on raising awareness amongst students about how much their peers actually drink (and do not drink) and to correct existing misperceptions [3]. Information about how much students actually consume, accurate statistics about the frequency of negative consequences among them and basic information relating to alcohol are part of the approach [4]. Currently there is no published Cochrane systematic

review on the effectiveness of social norms approaches, though a Cochrane protocol on this topic has been published [5]. One of the best trials we have identified so far has been by Kypri [6]in New Zealand, where an electronic Screening and Brief Intervention (SBI) approach identified students at high risk and then provided normative feedback to this group. After 12 months this group had significantly lower alcohol-related problem scores than controls. However, Kypri has not been able to establish whether the normative feedback intervention, or simply measuring drinking behaviour using the alcohol use disorders identification test (AUDIT) screening tool, was the active ingredient accounting for this effect. Kypri also suggested that a social desirability response bias may have influenced the results.

Potential population impact

Working with Kypri, we have used his dataset to model the potential of normative feedback to reduce alcohol related problems. Using a Bayesian approach [7], we have estimated that the change in alcohol related problem scores from Kypri's study might equate to a 5% prevalence reduction in alcohol disorders (DSM dependence and abuse), which would be a marked and important consequence [8]. We have not been able to assess impact on acute harms, violence and crime because of insufficient information for modelling. We are also interested in how the prevention paradox should inform the choice of intervention population. Although Kypri [9] and others have targeted high risk drinkers, the prevention paradox states that more harm comes from those at lower levels of risk, and Rossow [10] has recently demonstrated that this paradox holds, albeit to a lesser extent, for heavy episodic drinking and acute harms. Therefore it is possible that this relatively cheap intervention may be relatively more effective if delivered at a whole population level rather than just to those at higher levels of risk.

Methods/Design

Aims of the project

The aim of the trial is to determine the effectiveness of an on-line personalized feedback intervention for reducing alcohol consumption amongst undergraduate University students when compared with a control group, in both the UK and Portugal.

The objectives of this trial are:

• To examine the effectiveness of brief personalized normative feedback in reducing alcohol related problems in first and second year university undergraduate students

• To compare the effectiveness of brief personalized normative feedback between students in England and students in Portugal

• To assess the relative effectiveness of whole population (universal) versus screening and brief intervention (SBI; targeted) normative feedback in reducing alcohol related problems.

Design

A Solomon Three Group Design [11] will be used in each country (UK and Portugal) where participants will be randomly assigned, with concealed allocation, to one of three groups (see Figure 1). Baseline alcohol use and misuse will be measured in two of the groups, but not the third group. Demographic questions will be answered by all

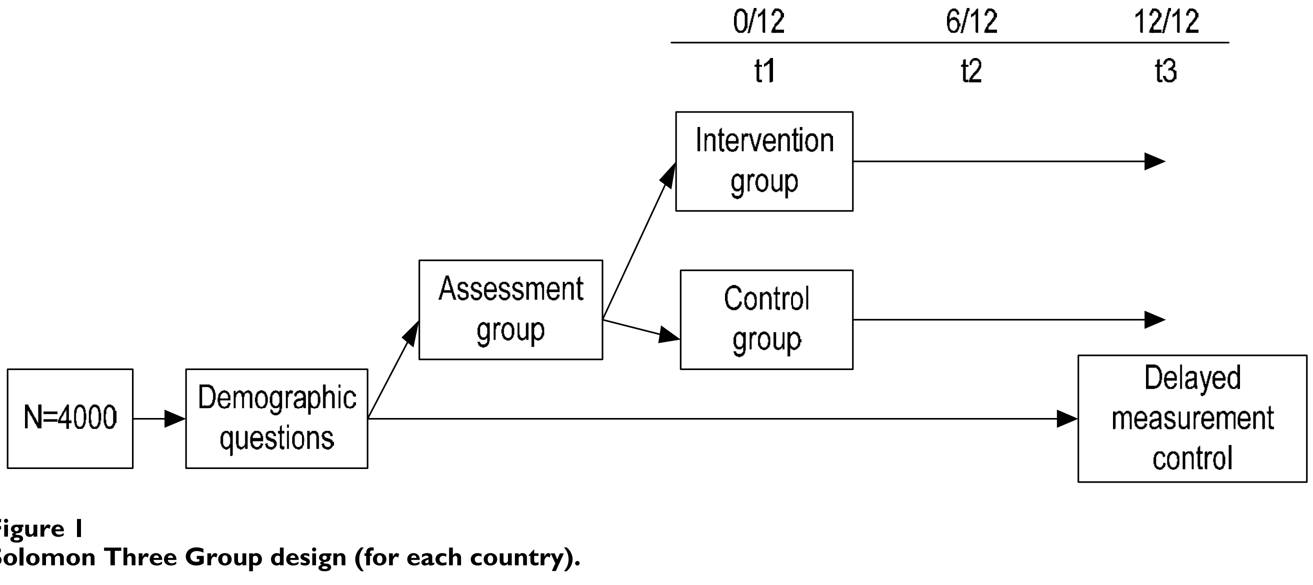

Figure 1
Solomon Three Group design (for each country).

three groups before randomization. There is one intervention group and two control groups, controlling separately for measurement and for intervention effects. The intervention group will receive the brief personalized normative feedback via email within a few weeks of completing the assessment and will be followed up at 6 months, along with the first control group. All three groups will be followed up at 12 months.

Ethics
Ethics approval for this study is provided by Oxford Brookes University Research Ethics Committee (REC No-2006/28).

Setting and participants
Participants are undergraduate university students (first and second year) enrolled in UK and Portuguese universities.

Recruitment
Undergraduate students in year one and two of their course will be invited to participate via poster, flyer, email or via university student information systems at the beginning of the academic year 2007/8. Oxford, Oxford Brookes, Nottingham and Plymouth Universities in the UK and Portucalense, Lusiada, Fernando Pessoa, Autonoma and ISMAI Universities from Portugal have agreed to collaborate. Furthermore, students will also be recruited via Facebook, a social networking website.

Randomisation
Randomisation occurs after students have read the consent form, with affirmative consent given proceeding to the on-line questionnaire. At this point, participants are randomised to intervention or control groups. Randomisation is achieved by concealed centrally-allocated computer generated random numbers.

Intervention
The brief personalised normative feedback given to each intervention group participant will comprise the results of their drinking behaviour assessment alongside information about alcohol and how it might affect them at their current drinking levels. The feedback will also compare their drinking – in graphical format – to the drinking of their student peers. Information will also be provided on the money that they might be spending on alcohol and also the calories they might be consuming at their current drinking levels. Details of sensible drinking levels will also be provided and contact details of health services and helplines they can contact if they feel they need further help.

Outcome measures
Alongside demographic questions (including: age, gender, weight, nationality, university year) we have carefully selected validated measurement instruments:

• Alcohol Use Disorders Inventory Test (AUDIT) and brief version (AUDIT-C)

• Alcohol Expectancies Questionnaire (AEQ, brief version)

• Social Desirability Scale (SDS, brief version)

• Self-report measures on alcohol consumption (from ESPAD and Centre for Addiction and Mental Health (CAMH), Toronto) [12,13]

• Perceived norms (validated adaptation of the two versions of the Drinking Norms Rating Form)

Data collection
Data are collected at baseline, six months and 12 months. All data collection is done online, through the trial website, accessed via a web-link promoted in all forms of advertisement.

A further methodological challenge is the expected high rate of attrition from follow-up. We have incorporated a number of features designed to improve this, including:

• Three e-mail reminders at seven day intervals to non-responders;

• The use of an appropriate incentive;

• Assurance of confidentiality.

Sample size calculation
A figure of 150 students per group is specified for analysis for both male and female hazardous drinkers resulting in an overall sample size requirement of 4000 students in each country. We intend to assess intervention efficacy in the student population as a whole and in particular a subgroup of hazardous drinkers (AUDIT $\geq$ 8), for both males and females. This group is likely to be a minority so the sample size has to be increased to ensure sufficient numbers of this high risk group to enable robust statistical analysis with power =.9 and α = .05 (2-tailed tests) and taking account of expected participation and attrition rates.

Analyses
Statistical tests of difference in proportions or mean difference tests (or non-parametric equivalents) will be used to test differences between intervention and control groups.

Multivariate analyses will be conducted with gender, nationality, University year (one and two), social desirability and study country as covariates. Data will be analysed by a researcher blinded to experimental group.

Discussion

This on-line randomised controlled trial has the potential to address three key issues. Firstly, evidence of the potential to reduce hazardous and harmful drinking amongst University students might lead to better prevention programmes across Europe. Second, evidence regarding the differential effectiveness of social norms interventions in countries with different drinking cultures and patterns. And last, evidence about whether universal or targeted approaches are better with this population group.

List of abbreviations used

AUDIT Alcohol Use Disorders Identification Test

RCT Randomised Controlled Trial

UK United Kingdom

ESPAD European School Survey Project on Alcohol and Other Drugs

CAMH Centre for Addiction and Mental Health

SDS Social Desirability Scale

AEQ Alcohol Expectancies Questionnaire

SBI Screening and Brief Intervention

DSM Diagnostic and Statistical Manual of Mental Disorders

Competing interests

The author(s) declare that they have no competing interests.

Authors' contributions

Both authors have contributed to the development of this protocol. TM is a research student supervised by DF. Both authors have contributed to the analytical strategy, on developing the intervention and overall methodological development. TM wrote the first and final drafts of the protocol. DF has contributed to the drafting process. Both authors have read and approved the final manuscript.

Acknowledgements

The trial is jointly funded by Fellowship and project grants from the Foundation for Science and Technology (Fundação para a Ciência e a Tecnologia [FCT]) (Portugal), the Alcohol Education and Research Council (AERC) (UK) and the European Research Advisory Board (ERAB) (Belgium). No funder has had any role in the preparation of the manuscript or the decision to submit.

References

1. WHO: **Global Burden of Disease study.** Geneva: World Health Organisation; 2001.
2. Jernigan D: **Global Status Report: Alcohol and Young People.** Geneva: World Health Organization; 2001:57.
3. Walters ST: **In praise of feedback: an effective intervention for college students who are heavy drinkers.** *Journal of American College Health* 2000, **48:**235-238.
4. Perkins HW: **Social Norms and the Prevention of Alcohol Misuses in Collegiate Contexts.** *Journal of Studies on Alcohol* 2002:164-172.
5. Moreira T, Foxcroft D: **Social norms interventions to reduce alcohol misuse in University or College students (Protocol).** *Cochrane Database of Systematic Reviews* 2007:CD006748.
6. Kypri K, Langley J, Saunders J, Cashell-Smith M: **Assessment may conceal therapeutic benefit: findings from a randomized controlled trial for hazardous drinking.** *Addiction* 2007, **102(1):**62-70.
7. Spiegelhalter D, Myles J, Jones D, Abrams K: **An introduction to bayesian methods in health technology assessment.** *BMJ* 1999, **319:**508-512.
8. Foxcroft D, Kypri K, Simonite V: **Bayesian estimates of population prevalence using the AUDIT diagnostic test for alcohol abuse and dependence.** *Annual Symposium of the Society for the Study of Addiction 2007; York* 2007.
9. Kypri K, Saunders J, Williams S, McGee R, Langley J, Cashell-Smith M, Gallagher S: **Web-based screening and brief intervention for hazardous drinking: a double-blind randomized controlled trial.** *Addiction* 2004, **99(11):**1410-1417.
10. Rossow I, Romelsjo A: **The extent of the 'prevention paradox' in alcohol problems as a function of population drinking patterns.** *Addiction* 2006, **101(1):**84-90.
11. Campbell D, Stanley J: *Experimental and quasi-experimental designs for research* Boston: Houghton Mifflin; 1963.
12. Barbro A, Björn H, François B, Marie C, Anna K, Anastasios F, Sabrina M, Alojz N, Januzs S, Marcis T: **Alcohol and Drug Use Among European 17–18 Year Old Students. Data from the ESPAD Project.** Stockholm: Sweden.: The Swedish Council for Information on Alcohol and Other Drugs (CAN) and the Pompidou Group at the Council of Europe; 2007:156.
13. CAMH: **Centre for Addiction and Mental Health.** *Canada* 2008 [http://www.camh.net/About_Addiction_Mental_Health/Drug_and_Addiction_Information/evaluate_your_drinking.html].

Pre-publication history

The pre-publication history for this paper can be accessed here:

http://www.biomedcentral.com/1471-2458/8/113/prepub

Publish with **BioMed Central** and every scientist can read your work free of charge

"BioMed Central will be the most significant development for disseminating the results of biomedical research in our lifetime."

Sir Paul Nurse, Cancer Research UK

Your research papers will be:

- available free of charge to the entire biomedical community
- peer reviewed and published immediately upon acceptance
- cited in PubMed and archived on PubMed Central
- yours — you keep the copyright

Submit your manuscript here:
http://www.biomedcentral.com/info/publishing_adv.asp

BioMedcentral
